# Knowledge and Attitude regarding COVID-19 among Pregnant Women in Southwestern Iran in the Early Period of its Outbreak: A Cross-Sectional Study

**DOI:** 10.4269/ajtmh.20-0608

**Published:** 2020-10-27

**Authors:** Najmeh Maharlouei, Nasrin Asadi, Khadijeh Bazrafshan, Shohreh Roozmeh, Abbas Rezaianzadeh, Mohammad-hassan Zahed-roozegar, Fatemeh Shaygani, Ali Kharmandar, Behnam Honarvar, Camellia Hemyari, Navid Omidifar, Marziyeh Zare, Kamran B. Lankarani

**Affiliations:** 1 Health Policy Research Center, Institute of Health, Shiraz University of Medical Sciences, Shiraz, Iran;; 2 Maternal-Fetal Medicine Research Center, Shiraz University of Medical Sciences, Shiraz, Iran;; 3 Colorectal Research Center, Shiraz University of Medical Sciences, Shiraz, Iran;; 4 Non-Communicable Disease Research Center, Fasa University of Medical Sciences, Fasa, Iran;; 5 Department of Pathology, Medical Education Research Center, Shiraz University of Medical Sciences, Shiraz, Iran

## Abstract

Both knowledge and attitude can play key roles in the prevention of novel COVID-19. This cross-sectional study was conducted on a statistical sample of pregnant women in southwestern Iran between March and April 2020 to evaluate their knowledge and attitude toward this condition. So, pregnant mothers registered in antenatal clinics affiliated to Shiraz University of Medical Sciences were called and asked to fill in a three-part online questionnaire including sociodemographic characteristics, obstetric/medical history, and knowledge/attitude toward COVID-19. *P*-values < 0.05 were considered statistically significant. The mean score of knowledge among 540 respondents was 34 (±4.1) out of 43. Also, 44.3% answered more than 80% of the items correctly. Higher knowledge scores were accordingly associated with marriage duration, area of residence, health insurance coverage, socioeconomic status (SES), and self-rated health status. However, a strong relationship was found between knowledge, SES, and health insurance coverage with reference to multivariate analysis results. Moreover, majority of the pregnant women and their households expressed their concern about using preventive measures against COVID-19. Although most respondents were moderately worried about becoming infected with COVID-19, 264 (48.9%) cases reported that they were very much anxious about their newborns being infected with COVID-19 and 388 (71.9%) individuals asserted that they were worried about their mortality due to this infection. Besides, most mothers maintained that they had some degrees of rumination, which could interfere with their routine daily chores. Hence, health policy-makers should pay much focus on educating pregnant mothers to help them prevent mental exhaustion.

## INTRODUCTION

Infectious diseases can play a significant role in pregnancy, particularly by affecting maternal and fetal outcomes.^1^ Prenatal respiratory infections may also result in stillbirth, miscarriage, and preterm delivery.^2^ Novel COVID-19 is a new respiratory infection that started from Wuhan Province in China and rapidly became widespread in all parts of the world, making the WHO call it “the pandemic of the century.”^3,4^ Accordingly, patients infected with COVID-19 would experience conditions ranging from a common cold to severe acute respiratory failure.^4^ In addition, no definite medication is still available to prevent or cure this disease, and preventive measures are the only methods to protect people against this infection.^5,6^

It is of note that pregnant women are more susceptible to developing severe cases of COVID-19 and consequently being admitted to hospitals and intensive care units because of their physiological changes.^7–10^ Although higher maternal age, presence of comorbidities, and high body mass index have been considered as risk factors for developing severe COVID-19 in expecting mothers.^10^ Moreover, one proven case of transplacental transmission of COVID-19 has been recorded thus far.^11^ SARS also belongs to the same family of COVID-19, with 25% case fatality rate during pregnancy and various perinatal complications, including disseminated intravascular coagulation, kidney (renal) failure, secondary bacterial pneumonia, sepsis, and abortion.^4^ Besides, the need for mechanical ventilation in pregnant mothers infected with SARS is higher than that for nonpregnant ones.^4^ According to the aforementioned conditions, prevention in the course of this outbreak is very vital for pregnant women. Because both knowledge and attitude can play key roles in prevention of infectious diseases,^12^ the present study was conducted on a statistical sample of pregnant women residing in Shiraz, the fifth populous city located in southwestern Iran, to figure out their knowledge and attitude toward COVID-19.

## MATERIALS AND METHODS

### Design and participants.

This cross-sectional study, approved by the Ethics Committee affiliated to Shiraz University of Medical Sciences (SUMS) and encoded IR.SUMS.REC.1398.1424, was fulfilled between March 24, 2020 and April 7, 2020 in the city of Shiraz, Iran. In addition, the Ethical Principles for Medical Research Involving Human Subjects, the World Medical Association Declaration of Helsinki was considered in this study.

For this purpose, pregnant women were recruited in this study if they were Iranian and had been living in the city of Shiraz for at least 6 months before the survey. The databases of all antenatal centers affiliated to SUMS were also used, where the personal information of nearly all mothers who would like to give birth to their babies in governmental hospitals had been registered. The personal information of such pregnant women included their first and last names and landline numbers and/or mobile phone numbers. The secretary of each center then called the pregnant mothers and explained the study and its objectives. Those women who agreed to participate received a link for filling in an online questionnaire. The ones who did not respond to their phones for three times or were unwilling to participate in the study were excluded. Approximately, a total number of 920 pregnant women were called, 842 cases agreed to fill in the questionnaire, and 678 mothers checked the site and browsed the questionnaires. Finally, 540 questionnaires were completely answered and submitted.

### Data collection instrument.

The data collection instrument in this study was an online self-administrated questionnaire, composed of three main parts: sociodemographic characteristics information, obstetric/medical history, and knowledge/attitude toward COVID-19. The first part of the questionnaire, that is, the sociodemographic information included respondents’ date of birth, date of marriage, area of residence (urban versus rural), level of education, occupation (stay-at-home versus employed), health insurance coverage, and socioeconomic status (SES) based on their claims, and perceived correlation between household income and expenditure. The second part of the questionnaire consisted of gestational age (GA), estimated date of delivery, number of pregnancies including the current one, comorbidities, and history of medication use including supplements. The third part was also a 24-item questionnaire to determine levels of knowledge and attitude among pregnant women toward COVID-19. The validity of this part was correspondingly approved by two community medicine specialists, two obstetricians, and one psychologist. The reliability was additionally acceptable (Cronbach’s alpha coefficient = 0.9%). Cronbach’s alpha coefficient for knowledge and attitude were, respectively, 91% and 85%.

The knowledge domain contained three items to assess the participants’ levels of knowledge in terms of the most common manifestations of COVID-19 (Knowledge-Q1), its routes of transmission (Knowledge-Q2), and serious symptoms making infected patients refer to hospitals (Knowledge-Q3). Within the knowledge domain, the respondents could also select many choices as they could think might be correct. Each correct answer was scored one, and each incorrect answer was scored zero. Furthermore, the last choice in each item was “I don’t know” which was scored zero. Therefore, the participants could, respectively, achieve scores of 0–14 for Knowledge-Q1, 0–15 for Knowledge-Q2, and 0–14 for Knowledge-Q3. The sum of Knowledge-Q1 to -Q3 was considered as knowledge regarding COVID-19, ranging between 0 and 43. As well, if the participants could obtain a knowledge score higher than 33 out of 43, used in binary logistic regression analysis, it would be considered as an acceptable level of knowledge.

Another section of the third part of the given questionnaire was a 21-item checklist evaluating the participants’ attitude toward COVID-19 in four main areas including preventive measures, concerns, and fears about COVID-19 affecting mothers and/or their newborns, effect of COVID-19 on maternity care, type of delivery and duration of lactation, and impact of quarantine on mothers’ mental health. This section was scored through a four-point Likert-type scale, 1 for “not at all,” 2 for “somewhat,” 3 for “moderately so,” and 4 for “very much so.” The final attitude score was the sum of scores of all attitude items, ranging between 21 and 84.

### Data analysis.

In this study, the data were analyzed using IBM SPSS Statistics software (version 18.0) (IBM Corp., Armonk, NY). The relationship between qualitative variables was further assessed via the chi-square test. To compare numerical variables between two groups, independent samples *t*-test and for the ones among three groups, analysis of variance were used. The variables with *P*-values less than 0.2 in univariate analysis were also entered into the logistic regression model, and backward elimination (alpha-to-remove = 0.1) was consequently used. *P*-values less than 0.05 were considered statistically significant.

## RESULTS

A total number of 540 pregnant women participated in this study, with age ranging from 17 to 49 (median = 31) years. The mean duration of their marriage was 7.7 years (±5.3), ranging from 1 to 28 years. Median GA was also 29 (7–40) weeks, and the mean was 27.8 (±8.1) weeks. As well, mean score for self-rated health status (SRHS) was 6.2 (±1.9), ranging from 1 to 10.

Table 1 shows the details of maternal comorbidities and history of medication use. The most common comorbidities were hypothyroidism (*n* = 42, 7.8%), diabetes mellitus (DM), and gestational DM (*n* = 31, 5.7%). On the other hand, one participant (0.2%) reported idiopathic thrombocytopenic purpura, as the least reported comorbidity. Regarding the history of medication use by mothers, the most common medication taken by 76 (14.1%) cases was ferrous sulfate and the second one was folic acid (*n* = 59, 10.9%). Detailed information in this respect is listed in Table 1.

**Table 1:** Pregnant women’s comorbidity and medication use history

Comorbidity	Frequency (%)	Medication	Frequency (%)
Hypothyroidism	42 (7.8)	Ferrous sulfate	76 (14.1)
Diabetes mellitus/GDM	31 (5.7)	Folic acid	59 (10.9)
Other endocrine disorders	20 (3.7)	Multi-vitamin	45 (8.3)
Headache	19 (3.5)	Levothyroxine	42 (7.8)
HTN	17 (3.1)	Aspirin	42 (7.8)
CVD	9 (1.7)	Other	57 (10.6)
Renal diseases	9 (1.7)	–	–
Respiratory diseases	7 (1.3)	–	–
Seizure	7 (1.3)	–	–
Idiopathic thrombocytopenic purpura	1 (0.2)	–	–

GDM = gestational diabetes mellitus; HTN = hypertension; CVD = cardiovascular diseases.

Table 2 presents the pregnant mothers’ knowledge regarding COVID-19. The first item was about the most common symptoms of COVID-19, the mean score of which was 10.8 (±1.7), and a total of 355 (65.7%) participants achieved 11 or higher out of 14. The second item was associated with routes of transmission. In this item, the mean score was 12.7 (±2.6), and 438 (81.1%) respondents gained 11 or higher out of 15, and just one case (0.2%) answered less than 30% of the items correctly. The third item was on the subject of serious symptoms making infected patient refer to hospitals. In this part, the mean score was 10.4 (±1.6), and 294 (54.4%) participants answered > 70% of the items correctly. The knowledge score related to COVID-19 (sum of the three previously mentioned knowledge items), ranging from 20 to 41. The mean score of knowledge was also by 34 (±4.1), and 239 participants (44.3%) answered more than 80% of the items correctly. As well, 253 pregnant mothers (46.9%) had a moderate level of knowledge (correct answer was between 65% and 80%) regarding COVID-19, although none of the participants achieved less than 20 out of 43.

**Table 2:** Pregnant women’s knowledge regarding COVID-19

Knowledge regarding				
Q1: Most common symptoms of COVID-19*				
Mean (±SD)	Median (minimum–maximum)	0–5 *f* (%)	6–10 *f* (%)	11–14 *f* (%)
10.8 (±1.7)	11 (3–14)	4 (0.7)	181 (33.5)	355 (65.7)
Q2: Route of transmission of COVID-19†				
Mean (±SD)	Median (minimum–maximum)	0–5 *f* (%)	6–10 *f* (%)	11–15 *f* (%)
12.7 (±2.6)	14 (5–15)	1 (0.2)	101 (18.7)	438 (81.1)
Q3: Alarming symptoms infected patients with COVID-19 should refer to hospital‡				
Mean (±SD)	Median (minimum–maximum)	0–5 *f* (%)	6–10 *f* (%)	11–14 *f* (%)
10.4 (1.6)	11 (3–12)	4 (0.7)	242 (44.8)	294 (54.4)
Summary of Q1–Q3: Knowledge regarding COVID-19§				
Mean (±SD)	Median (minimum–maximum)	20–27 *f* (%)	28–35 *f* (%)	36–43 *f* (%)
34 (±4.1)	35 (20–41)	48 (8.9)	253 (46.9)	239 (44.3)

* Score ranged between 0 and 14.

† Score ranged between 0 and 15.

‡ Score ranged between 0 and 14.

§ Score ranged between 0 and 43.

Table 3 compares the knowledge score regarding COVID-19 based on the participants’ SES and obstetric/medical history. Accordingly, the knowledge score was the highest in mothers who had been married for more than 10 years, which was significantly higher than those being married for 5–10 years (*P*-value = 0.02). Pregnant women who had been residing in urban areas had also significantly achieved higher knowledge scores than their rural counterparts (*P*-value = 0.001). As well, 52 participants (9.6%) were employed; however, the mean score of knowledge was significantly higher in employed individuals than stay-at-home mothers (35 versus 33.9, *P*-value = 0.03). Moreover, 222 pregnant mothers (41.1%) had a university degree, who had obtained significantly higher knowledge score regarding COVID-19 than those with lower levels of education (*P*-value = 0.01). Most participants had health insurance coverage (435; 80.6%), and they had significantly higher levels of knowledge than mothers with no insurance coverage (*P*-value < 0.001). With reference to the correlation between household income and expenditure, 136 pregnant women (25.1%) had reported their income equal to their expenditure, 401 mothers (74.3%) had reported their expenditure more than their income, and three cases (0.6%) had reported it vice versa. The mean score of knowledge was significantly the highest in cases whose income was more than their expenditure (*P*-value = 0.04). Regarding SES, a total number of 242 (44.8%), 256 (47.4%), and 42 (7.8%) mothers had asserted that they belonged to low, middle, high SES. There was also a significant difference between the mean score of knowledge in high- and low-SES groups (*P*-value = 0.006). The pregnant women had further rated their health status as poor (*n* = 122, 22.6%), moderate (*n* = 257, 47.6%), and good (*n* = 242, 44.8%). It should be noted that the mean score of knowledge was significantly lower in the group with moderate SRHS (*P*-value = 0.03).

**Table 3:** Knowledge score based on pregnant women’s demographic characteristics and SES

Maternal information		Knowledge score	
Maternal age (years)	Frequency (%)	Mean (±SD)	*P*-value
< 19 odds ratio > 34	169 (31.3)	33.5 (±4.5)	0.13
18 < age < 35	371 (68.7)	34.2 (±3.8)	
Marriage duration (years)			
1–5	232 (43)	34 (±3.9)	0.02*
6–10	168 (31.1)	33.3 (±4.4)	
> 10	140 (25.9)	34.6 (±3.8)	
Number of pregnancies			
First pregnancy	195 (36.2)	34 (±3.8)	0.9
Second pregnancy	166 (30.7)	34 (±4.2)	
Third or more pregnancy	179 (33.1)	33.9 (±4.3)	
Gestational age (weeks)			
< 14	36 (6.7)	33.3 (±4.5)	0.7
14–28	184 (34.1)	34.2 (±3.9)	
> 28	320 (59.3)	33.9 (±4.1)	
Area of residence			
Urban	425 (78.7)	34.3 (±3.9)	0.001
Rural	115 (21.3)	32.8 (±4.4)	
Occupation			
Stay-at-home	488 (90.4)	33.9 (±4.1)	0.03
Employed	52 (9.6)	35 (±3.5)	
Highest educational attainment			
Below high school diploma	119 (22)	33.1 (±4.2)	0.01*
High school diploma	199 (36.9)	33.9 (±4.1)	
University degree	222 (41.1)	34.5 (±3.9)	
Health insurance coverage			
Insured	435 (80.6)	34.4 (±3.9)	< 0.001
Noninsured	105 (19.4)	32.3 (±4.4)	
Correlation between income and expenditure			
Equal	136 (25.1)	34.1 (±3.9)	0.04†
Expenditure > income	401 (74.3)	33.9 (±4.1)	
Income > expenditure	3 (0.6)	37.3 (±2.5)	
Claimed SES			
Low	242 (44.8)	33.4 (±4.3)	0.006*
Middle	256 (47.4)	34.4 (±3.8)	
High	42 (7.8)	34.7 (±3.9)	
SRH			
Poor	122 (22.6)	34.9 (±4)	0.03‡
Moderate	257 (47.6)	33.5 (±4.2)	
Good	242 (44.8)	34.4 (±3.8)	
Presence of comorbidities			
No comorbidity	452	33.9 (±4.1)	0.4
One comorbidity	58	34.5 (3.2)	
> 1 comorbidities	30	34.3 (±4.9)	

SES = socioeconomic status.

* Mean knowledge score was significantly different between the first and the third group.

† Mean knowledge score was significantly different between the third and the first two groups.

‡ Mean knowledge score of the second group was significantly different with the other two groups.

As shown in Table 4, the results of logistic regression analysis revealed that SES and health insurance coverage were associated with achieving acceptable knowledge scores (> 33 out of 43). Therefore, the higher the SES, the higher was the number of participants obtaining acceptable knowledge scores. Moreover, pregnant mothers who had health insurance coverage had 3.4 times higher chance of gaining acceptable knowledge scores (*P*-value < 0.001, CI for odds ratio: 2.1–5.3).

**Table 4:** Determinants of achieving acceptable knowledge score (> 75% of total score)

	OR	95% CI for OR	*P*-value
Socioeconomic status	–	–	0.01
Low	1	–	–
Intermediate	1.7	1.1–2.5	0.012
High	2.4	1.1–5.8	0.047
Having insurance			< 0.001
No	1	–	–
Yes	3.4	2.1–5.3	< 0.001

OR = odds ratio. Acceptable knowledge: if score was > 33 (out of 43).

Table 5 illustrates the pregnant women’s attitude toward COVID-19. In this regard, majority of the participants reported themselves (491; 90.9%) and their households (*n* = 463, 85.7%) were extremely concerned about preventive measures against COVID-19. With regard to being at risk of COVID-19, 74 (13.7%) mothers had reported “not at all,” 249 (46.1%) cases had reported “somewhat,” 140 (25.9%) individuals had reported “moderately so,” and 77 (14.3%) cases had reported “very much so.” Although 43 pregnant women (8%) were not anxious at all, others had somewhat (*n* = 179, 33.1%), moderate (*n* = 123, 22.8%), or very much (*n* = 195, 36.1%) anxiety about being infected with COVID-19. A total number of 264 pregnant women (48.9%) were also so much worried about their newborns being infected with COVID-19, whereas 66 (12.2%) cases were not worried at all. Most mothers (*n* = 388, 71.9%) were extremely anxious about their newborns’ mortality and morbidity caused by COVID-19, and 480 pregnant women (88.9%) had significantly reduced their face-to-face communications. Besides, 271 pregnant mothers (50.2%) had reported that their routine prenatal care had been diminished or even discontinued because of closure of antenatal clinics, and 50 (9.3%) women had continued their routine prenatal care in accordance with the timetable set. On the other hand, stress regarding being infected with COVID-19 had made most mothers (*n* = 388, 71.9%) remarkably reduce or discontinue their routine prenatal care. All the participants had also reported degrees of obsession with washing hands from low (*n* = 223, 41.3%) to high (*n* = 166, 30.7%) degrees. In addition, all the pregnant mothers believed that they had been successful in controlling their stress about COVID-19, but at different levels. Details regarding mothers’ attitude toward COVID-19 are shown in Table 5.

**Table 5:** Pregnant women’s attitude toward COVID-19

		Not at all, *f* (%)	Somewhat, *f* (%)	Moderately so, *f* (%)	Very much so, *f* (%)
A1	You are concerned about taking preventive measures against COVID-19	1 (0.2)	3 (0.6)	45 (8.3)	491 (90.9)
A2	Your households are concerned about taking preventive measures against COVID-19	1 (0.2)	7 (1.3)	69 (12.8)	463 (85.7)
A3	You consider yourself at risk of COVID-19	74 (13.7)	249 (46.1)	140 (25.9)	77 (14.3)
A4	You are anxious about being infected with COVID-19	43 (8)	179 (33.1)	123 (22.8)	195 (36.1)
A5	You are worried about being infected with COVID-19 during delivery or postpartum hospital stay	32 (5.9)	89 (16.5)	100 (18.5)	319 (59.1)
A6	You are worried about your newborn being infected with COVID-19	66 (12.2)	132 (24.4)	78 (14.4)	264 (48.9)
A7	You are anxious about your newborn’s mortality by COVID-19	11 (2)	52 (9.6)	89 (16.5)	388 (71.9)
A8	Your routine prenatal care (physical and para-clinic examinations) has been reduced or discontinued due to closure of antenatal clinics	50 (9.3)	87 (16.1)	132 (24.4)	271 (50.2)
A9	Stress regarding being infected with COVID-19 makes you reduce or discontinue your routine prenatal care (physical and para-clinic examinations)	24 (4.4)	49 (9.1)	79 (14.6)	388 (71.9)
A10	COVID-19 outbreak will affect your type of delivery (natural, i.e., vaginal delivery or cesarean section)	85 (15.7)	145 (26.9)	116 (21.5)	194 (35.9)
A11	COVID-19 outbreak will negatively affect your newborn’s exclusive breastfeeding duration	80 (14.8)	164 (30.4)	123 (22.8)	173 (32)
A12	Concerns about being infected with COVID-19 have reduced your face-to-face communications with others	8 (1.5)	19 (3.5)	33 (6.1)	480 (88.9)
A13	You receive emotional support from your households and your social networks	0	51 (9.4)	100 (18.5)	389 (72)
A14	You follow the news about COVID-19 on social media	0	85 (15.7)	137 (25.4)	318 (58.9)
A15	Following news regarding COVID-19 makes you anxious and upset	0	81 (15)	147 (27.2)	312 (57.8)
A16	COVID-19 has negatively affected your routine daily chores	0	127 (23.5)	223 (41.3)	190 (35.2)
A17	You are dealing with rumination regarding COVID-19 consequences	0	245 (45.4)	167 (30.9)	128 (23.7)
A18	You feel obsessed with washing hands and disinfecting objects	0	223 (41.3)	151 (28)	166 (30.7)
A19	Home quarantine and social distancing have negatively affected your mood	0	171 (31.7)	166 (30.7)	203 (37.6)
A20	Quality and quantity of your sleep have been negatively affected by COVID-19	0	281 (52)	138 (25.6)	121 (22.4)
A21	You have been successful to control your stress about COVID-19	0	69 (12.8)	207 (38.3)	264 (48.9)

## DISCUSSION

In this study, knowledge and attitude toward COVID-19 were evaluated among pregnant women residing in the city of Shiraz, Iran, between March 24 and April 7, 2020. The findings revealed that 70% of the participants had an acceptable level of knowledge in this regard. Although the highest knowledge score was about routes of transmission of COVID-19, the least score was achieved on serious symptoms making infected patients refer to hospitals. Higher knowledge score was also associated with marriage duration, area of residence (urban versus rural), health insurance coverage, SES, and SRHS. However, a strong relationship was observed between knowledge, SES, and health insurance coverage with reference to multivariate analysis results.

In the second part of the study, the findings correspondingly demonstrated that majority of the pregnant mothers and their households were concerned about using preventive measures against COVID-19. Although most respondents were moderately worried about becoming infected with COVID-19, about half of them reported that they were very anxious about their newborns being infected with COVID-19, and two-thirds stated that they were worried about their newborns’ mortality due to this infection. The mentioned concerns had further made majority of these pregnant mothers reduce or discontinue their routine prenatal care. It should be noted that lack of routine prenatal care might thus increase adverse pregnancy outcomes in various ways.^13,14^ Failure in early detection of abnormal fetuses would additionally lead to loss of the legal window for abortion. No timely diagnosis and treatment of gestational DM and hypertension can also have adverse effects on both maternal and fetal conditions.^15^ In addition, proper assessment of maternal weight gain, following fetal growth, vaccination, timely Rh_o_(D) immune globulin (Rh_o_GAM) shot in special conditions, etc. could be missed in this situation. Besides, most mothers maintained that they had some degrees of rumination, interfering with their routine daily chores.

The results of this study indicated that the overall knowledge regarding COVID-19 was poor in less than 9% of pregnant women, although about 70% of the cases achieved acceptable knowledge score. These findings were supported by surveys conducted in different countries, for example, Nigeria, China, the United States, and Bangladesh, suggesting that pregnant women had acceptable levels of knowledge of COVID-19.^16–19^ This could be the result of efforts made by governmental and nongovernmental organizations aimed to educate people through various methods including social media, newspapers, television programs, and short message services. However, these findings were not universal as Sirchan et al.^20^ had reported that most Thai women (74.1%) had poor knowledge about COVID-19.

Talking about knowledge, the highest knowledge score was found for routes of transmission of COVID-19, which had been correctly answered by more than 80% of the pregnant mothers. These findings were consistent with the results of the study by Farhanan and Mannan^17^ in which most Bangladeshi women had adequate knowledge about routes of transmission of COVID-19. This high level of knowledge can be considered as an advantageous point because it can lead people to take proper preventive measures. Besides, about two-thirds of the participants in the present study had acceptable knowledge regarding the most common manifestations of COVID-19. Having an acceptable level of knowledge regarding common symptoms of this condition can help people become aware of common symptoms of diseases and on-time referrals to healthcare centers and hospitals, hence not infect unaffected individuals. Nevertheless, this domain needs special attention because it can act as a double-edged sword. If people refer to hospitals for any unimportant manifestations of COVID-19, it can result in burnout among healthcare workers. On the other hand, referring late to hospitals can give rise to higher rate of severe cases and even higher mortality rates.

Similar to the findings of the present study, Rahman and Sathi^21^ and Abdelhafiz et al.^22^ had revealed that individuals living in urban areas had more knowledge about COVID-19. This could be because of more access to information technologies. The given results were further supported by other surveys that had shown that occupation,^18,19,21^ level of education,^18,19,21^ and area of residence^18^ were predictors of knowledge score; hence, people with lower levels of education and unemployment had gained lower knowledge score.^18,19,21^ With reference to occupation, higher knowledge score could be because of more connections with other people, so there were more concerns about the virus, discussions about it with colleagues, and attempts to get more knowledge to protect themselves and their family. Moreover, Abdelhafiz et al.^22^ had demonstrated a direct correlation between knowledge score and income as reported in the present study. Based on logistic regression analysis results, SES and health insurance coverage could have significant roles in achieving acceptable knowledge score. This may happen as people with higher SES are supposed to have more access to information devices and have larger social networks. Besides, they generally have higher levels of education than those in lower SES groups.^23^ All things together may lead to having more knowledge about COVID-19 in individuals with higher SES. On the other hand, health insurance coverage in Iran is somehow related to SES. Based on insurance policy in Iran, an individual can have insurance through different methods. The most common method is being insured by employers either in governmental or in private sectors.^24^ Similarly, people who are self-employed can insure themselves by paying the premium on a monthly basis.^24^ In addition, stay-at-home individuals can insure themselves if they pay insurance fees and charges themselves.^24^ Accordingly, people with health insurance coverage can have more stable income, and this can lead to higher levels of education and access to more information technologies. Therefore, individuals benefiting from insurance can get more information about COVID-19 and reach acceptable knowledge score about it.

The study findings additionally showed that majority of the participants and their households were very much concerned about the preventive measures against COVID-19, in line with the results reported by Yassa et al.^25^ in which most Turkish pregnant women had taken adequate precautions against COVID-19. Furthermore, most participants in the present study maintained they that had reduced face-to-face communications, supported by results of the survey by Corbett et al.^26^ in Ireland. These findings also reflected on pregnant mothers’ attention to preventive instructions, which could be rooted in their higher degrees of concerns toward their own health status and that of their newborns.

The present study revealed that most pregnant women had reported various degrees of worry about becoming infected with COVID-19, consistent with the findings from two other surveys on COVID-19 among pregnant mothers residing in Shanghai, China, and in Istanbul, Turkey.^25,27^ The study findings also showed that most participants had moderate to high concerns about being infected with COVID-19 during delivery or postpartum hospital stay, far more than pregnant women in Istanbul, in which one-third of the participants had mentioned it as a hard situation.^25^

In contradiction of the present study in which majority of the participants were worried about their newborns’ infection with COVID-19, Yassa et al.^25^ had reported that less than half of pregnant mothers had this concern. However, concern regarding newborns’ mortality due to COVID-19 was relatively similar to that in the present study and in Corbett’s in Ireland.^26^ The high degree of concern toward morbidity and mortality associated with COVID-19 might be derived from a low global data about the nature of COVID-19 and lack of promising medical treatments*.*

In terms of prenatal care, three-quarters of the pregnant women in the present study reiterated that their routine prenatal care had been reduced or discontinued. The reasons were mothers’ concerns about becoming infected with COVID-19, as well as closure of antenatal clinics for nearly 2 months from March to April. Likewise, a study in Shanghai had reported that pregnant women, especially in the second trimester, were more willing to cut down the frequency of prenatal care and over 85% of the participants, particularly the primiparous ones, had requested online consultations to avoid being present in crowded places.^27^ This pointed to the participants’ knowledge and concerns regarding the routes of transmission of COVID-19, which could be through direct encounters with infected patients in crowded places or via contacts with infected surfaces.

Concerning the effect of COVID-19 on pregnant women’s mental health status, the study findings revealed that none of the participants was negligent regarding COVID-19 and its consequences. Although the degree of worry was different among the respondents, these negative thoughts had affected about one-third of Turkish pregnant women in Yassa’s survey.^25^ Moreover, similar to the results of the present study in which the outbreak had a detrimental effect on moods of most pregnant mothers, a cohort study in China had found an increasing trend in the prevalence of perinatal depression following the current outbreak.^28^ According to these findings, pregnant mothers were suffering from the mental burden of the COVID-19 pandemic because they would have been concerned about their own health status and that of their newborns.

Among the weaknesses of the present study was no access to participants from other provinces in Iran. Because Shiraz was one of the cities whose residents were following social distancing rules more strictly, the results cannot be also generalized to the whole country. In addition, the questionnaire was a self-administered online one, which needed more patience than those filled through face-to-face interviews. Therefore, some participants who checked the site link but not finished the survey were missed.

One of the most prominent strengths of this study was the time it was implemented. In fact, the present study was conducted when COVID-19 was at its peak in Iran. Moreover, a self-administered questionnaire was used in the present study by which pregnant women could feel more comfortable to complete it on their free time, which increased the accuracy of the answers.

In sum, although majority of the pregnant women in this study had an acceptable level of knowledge regarding the routes of transmission and serious symptoms of COVID-19, the lowest knowledge score was on serious symptoms making infected patients with COVID-19 refer to hospitals. Hence, health policy-makers should lay much focus on educating people regarding situations in which infected patients including pregnant mothers should refer to hospitals. In fact, unnecessary referrals would lead to burnout among healthcare workers, whereas late referrals would result in higher rates of morbidities and mortalities as well as increased expenditure imposed on healthcare systems. Besides, a strong correlation was found between the participants’ knowledge score and higher SES, which implicitly denoted the necessity of special attention to educating lower SES groups through different methods. Furthermore, the study participants had a remarkable worry regarding becoming infected with COVID-19, which could interfere with their routine daily chores. Therefore, more information should be given to pregnant mothers to prevent them from becoming mentally exhausted.
